# Limited Link of Common Blood Parameters with Tinnitus

**DOI:** 10.3390/jcm12113814

**Published:** 2023-06-01

**Authors:** Jan Bulla, Petra Brueggemann, Małgorzata Wrzosek, Sven Klasing, Benjamin Boecking, Laura Basso, Amarjargal Nyamaa, Stamatina Psatha, Matthias Rose, Birgit Mazurek

**Affiliations:** 1 Department of Mathematics, University of Bergen, 5020 Bergen, Norway; jan.bulla@uib.no; 2 Department of Psychiatry and Psychotherapy, University of Regensburg, 93053 Regensburg, Germany; 3 Tinnitus Center, Charité—Universitätsmedizin Berlin, Corporate Member of Freie Universität Berlin and Humboldt-Universität zu Berlin, 10117 Berlin, Germany; petra.brueggemann@charite.de (P.B.); sven.klasing@rbb-mueritz.de (S.K.); benjamin.boecking@charite.de (B.B.); laura.basso@charite.de (L.B.); nyamaa.amarjargal@charite.de (A.N.); stamatina.psatha@charite.de (S.P.); 4 Faculty of Psychology and Cognitive Science, Adam Mickiewicz University in Poznan, 60-568 Poznań, Poland; malgorzata.wrzosek@usz.edu.pl; 5 Medical Department, Clinic of Psychosomatic Medicine, Charité—Universitätsmedizin Berlin, Corporate Member of Freie Universität Berlin and Humboldt-Universität zu Berlin, 10117 Berlin, Germany; matthias.rose@charite.de

**Keywords:** tinnitus, blood parameters, erythrocytes, vitamin D3, uric acid, tinnitus questionnaire, tinnitus loudness, sensation level, hearing threshold

## Abstract

*Background*: Tinnitus severity is generally assessed by psychometric and audiological instruments. However, no objective measure exists to evaluate the subjective discomfort and suffering caused by this hearing phenomenon. The objective of this work was to determine the possible blood parameters for diagnostics and therapy. *Methods*: We measured tinnitus distress by using the Tinnitus Questionnaire (TQ) and collected tinnitus-related audiological measures, namely the hearing threshold (HT), tinnitus loudness (TL), and sensation level (SL, i.e., the tinnitus loudness/hearing threshold at a tinnitus frequency). Blood samples were taken from 200 outpatients of the Tinnitus Centre of the Charité, and 46 routine blood count parameters were examined. The possible interactions were determined by (robust) linear models. *Results*: Tinnitus distress and audiological measurements were largely uncorrelated but could partly be predicted by selected blood parameters. First, the erythrocyte counts predicted tinnitus distress to a small extent. Second, the levels of vitamin D3 explained about 6% of tinnitus loudness and, age-dependently, the hearing threshold variability. Last, the levels of uric acid explained about 5% of the sensation level variability. *Conclusions*: Tinnitus is a multidimensional phenomenon. The marginal influences of blood markers suggest the possible roles of inflammation and oxidative stress produced by psychological or somatic burdens. Clinically, a vitamin D substitution (in older patients) might have a hearing-protective effect.

## 1. Introduction

Tinnitus is a widespread phenomenon affecting at least 14% of the European population (14% in men and 15% in women [1]). This causes high health care costs, especially when tinnitus distress is present (severe tinnitus in Europe is 1–2%: 1.0% in men and 1.4% in women [1]). Tinnitus prevalence significantly increases with age and the worsening of a person’s hearing status. Tinnitus symptom severity is positively associated with healthcare resource use [1,2].

How exactly tinnitus occurs is still not well understood. Peripheral hearing damage is suspected, which brings about maladaptive changes in the central nervous system in a bottom-up process. In this process, non-auditory regions are involved as well. Accordingly, emotional and attention-related processing is relevant as well (see, e.g., [3,4]).

In a recent review, Deklerck and colleagues summarized the non-otologic risk factors for tinnitus [5]. When analyzing 55 studies, their bottom line was that primarily neuropsychiatric and orthopedic diseases correlate with an increased risk of tinnitus. Further, the authors considered 28 studies to evaluate the possible impact of cardiovascular risk factors, mainly hypertension, dyslipidemia, and ischemic heart and cerebral infarctions. No single factor showed a stringent connection with tinnitus—although it is referred to as an analysis, according to which tinnitus sufferers are more likely to suffer from hypertension and diabetes. This could, however, be the result of an increased general stress experience. Overall, it can only be assumed that multimorbidity is associated with an increased risk of tinnitus. Nevertheless, the consequences of tinnitus on daily life vary widely among individuals due to its associated co-morbidities, such as concentration or sleep difficulties and mental health problems [6].

New definitions differentiate between “tinnitus” as an auditory/sensory phenomenon and “tinnitus disorder” as the combination of the auditory component and associated suffering [7]. Accordingly, commonly measured outcome domains and questionnaires vary across studies. In a review [8], tinnitus loudness was the most frequently reported domain (14%), followed by tinnitus distress (7%), measured by a wide variety of questionnaires. Thus far, no single outcome or instrument for describing tinnitus has been established, which often impedes the comparability of research results.

A previous study of our research group investigated associations between blood parameters and perceived stress in the same sample of tinnitus patients [9]. In the current work, we use the same data, focusing on the possible links between blood parameters and three audiological measures, namely the hearing threshold (HT), tinnitus loudness (TL), and sensation level (SL), and the Tinnitus Questionnaire (TQ) [10] score, respectively. The aim of finding the biomarkers for hearing ability and tinnitus loudness or distress is an important topic in tinnitus research. We present a summary of studies conducted thus far on chronic subjective tinnitus and blood parameters in Table 1 (modified from [11]).

In this study, we classify the examined blood parameters into four major groups, three of which approximately correspond to the structure displayed in Table 1: (I) immune activity, (II) redox network activity (reduction/oxidation system), (III) metabolic activity, and additionally, (IV) telomere (for further information, see [24]).

**Figure 1:**
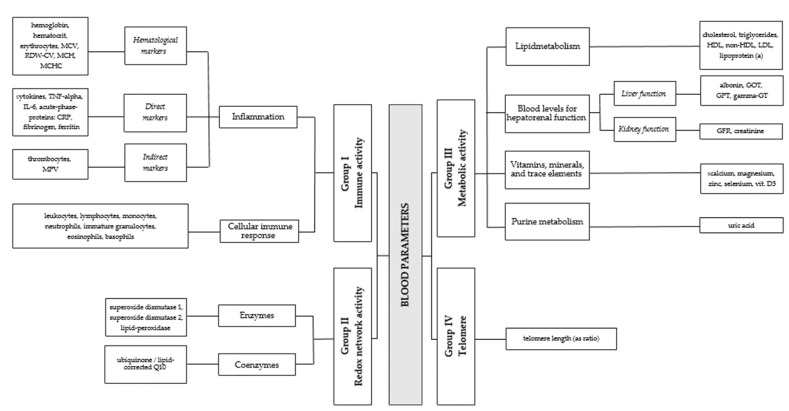
Chart of the four groups of blood markers considered in the present study.

We divided the immune activity group (Group I) into two large subgroups. The first subgroup reflects the cellular immune responses, and the second subgroup represents inflammation and can be divided into three further subgroups: *direct inflammation markers*, *indirect inflammation markers*, and *hematological markers*. The redox network activity (Group II) is also divided into two subgroups: (1) enzymes and (2) co-enzymes. Metabolic activity (Group III) is divided into four subgroups: (1) lipid metabolism, (2) blood levels for the hepatorenal function of *liver function* and *kidney function*, (3) purine metabolism, and (4) vitamins, minerals, and trace elements (cf. [9]). See Figure 1 for a visualization of this structure by means of an illustrative chart.

In recent years, biomarker studies have been conducted increasingly [11,25]. However, they often examine the link between tinnitus distress and the biological parameters without addressing the potentially overlapping (i.e., confounding) effects related to certain covariates, such as age and sex, hearing ability, tinnitus loudness, or sensation level. Since it is unclear which variable most closely reflects impairment due to tinnitus, we use both the psychologically oriented construct of tinnitus distress (measured by TQ) and the audiological measures of hearing threshold, tinnitus loudness, and sensation level as the outcome variables.

Thus, the research task at hand mainly aims at identifying possible tinnitus-related blood parameters and their potential link to the above-mentioned four outcome groups. Note, however, that tinnitus loudness and sensation level are co-determined by a person’s hearing ability. A reciprocal relationship between hearing ability and tinnitus distress is possible, but not assumed [3,26]. Hearing, in turn, deteriorates with age [27]. Last, age and sex are the co-determinants for the expression of certain blood parameters. The extent to which tinnitus is influenced by these variables and the role blood markers play needs to be clarified.

All blood parameters were collected once. Multiple analyses were performed: the determined blood parameters were correlated with the questionnaire scores measuring the tinnitus distress and audiological measures. We investigated whether certain blood parameters are linked to hearing loss, tinnitus loudness, sensation level, or tinnitus distress.

## 2. Materials and Methods

### 2.1. Sample and Blood Levels

**Figure 2:**
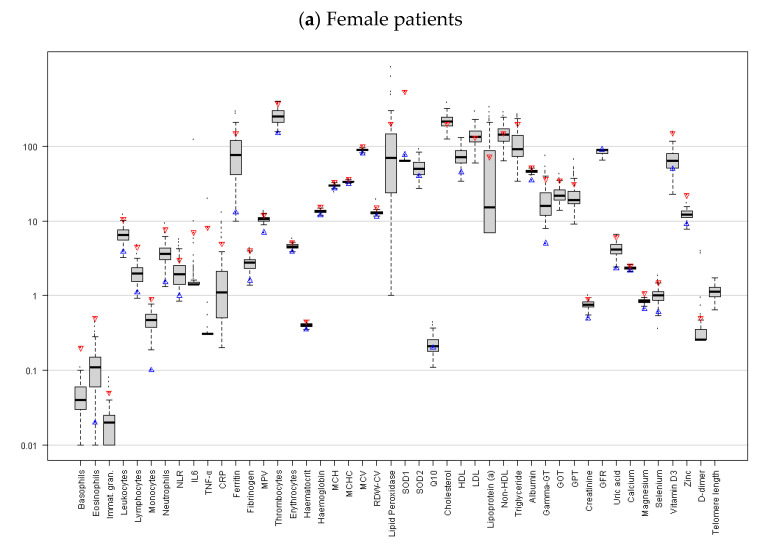

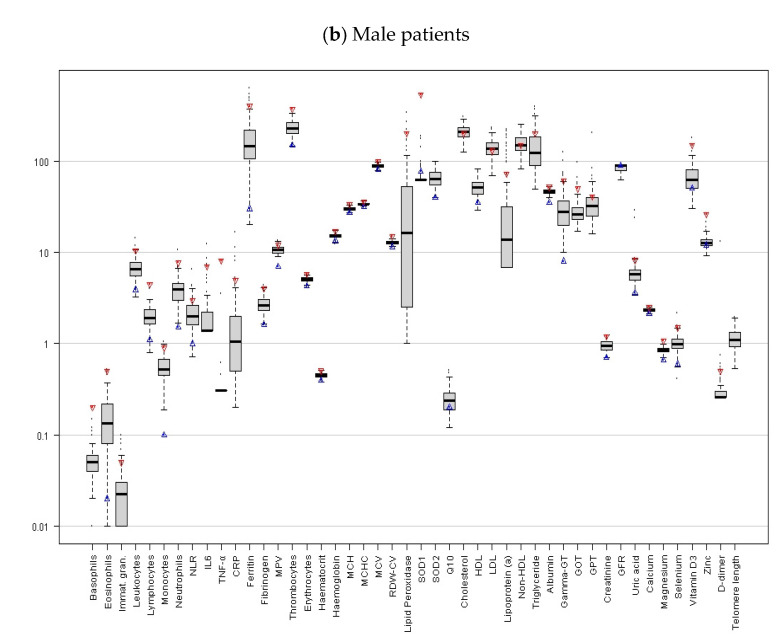
This figure shows box plots of blood parameter levels measured for female (upper panel (**a**)) and male (lower panel (**b**)) patients. Red and blue triangles represent the upper and lower bounds of reference ranges, as recommended by Labor Berlin [29].

Our study is based on the questionnaire data and blood markers from patients with chronic tinnitus (*N* = 200, 51% are female; the average age is 54.7 years). These patients self-referred to the Tinnitus Centre at Charité Universitätsmedizin Berlin between April 2016 and August 2017. The inclusion criteria included chronic tinnitus (i.e., lasting for more than 3 months), an age of 18 years or older, and having completed the Tinnitus Questionnaire and the Perceived Stress Questionnaire [28], along with other measures. The participants provided blood samples upon arrival at the Tinnitus Centre. These were obtained via 1 × 2 mL, 1 × 6 mL EDTA, 2 × 4.5 mL lithium heparin, 2 × 4.5 mL serum, and 1 × 2.7 mL citrate tubes. Blood samples were taken from a peripheral vein before the commencement of therapy. Subsequently, the above-mentioned blood parameters were then determined by the Berlin laboratory or the Biovis Diagnostik MVZ laboratory (see Figure 2 and Table 2 for a visualization and descriptive overview, respectively).

### 2.2. Tinnitus Questionnaire and Audiological Measurements

For the measurement of tinnitus distress, we relied on the German version of the Tinnitus Questionnaire. This questionnaire was selected to ensure comparability with previous studies that were carried out by our working group. The Tinnitus Questionnaire comprises 40 items with three response categories (2 = “agree”, 1 = “partially agree”, 0 = “disagree”). Patients’ hearing thresholds were measured using pure tone audiometry alongside the tinnitus frequency, tinnitus loudness (dB HL), and sensation level (dB SL). In detail, for pure tone audiometry, the measurements were performed binaurally for the frequency range between 0.25–8 kHz at eight steps (0.25 kHz, 0.5 kHz, 1 kHz, 2 kHz, 3 kHz, 4 kHz, 6kHz, and 8kHz). The hearing threshold (variable HT) was determined by increasing the decibels (dB) in the measured frequency ranges. The hearing threshold (in dB) is the minimal threshold at which a tested individual perceives the sound wave of the presented sinusoidal tones. For this study, we calculated the average hearing threshold across all frequencies [30,31].

Tinnitus matching is an audiological examination used to approximate the psychoacoustic tinnitus parameters. Tinnitus matching was carried out by the ENT specialist, who adjusted the sounds presented to the patients via headphones in such a way that the sounds were as close as possible to the type and frequency of sound and the subjectively perceived tinnitus level. For the remainder of this paper, the subjectively perceived tinnitus level is denoted as tinnitus loudness and corresponds to the variable TL (in dB). The sensation level (variable SL) is calculated from the hearing threshold (also denoted as hearing ability) in the tinnitus frequency minus the tinnitus loudness.

Table 3 summarizes our sample characteristics and questionnaires. Note that about 31% of the observations are missing for TL and SL because a measure could not be obtained due to non-tonal tinnitus. The additional variable TF denotes the tinnitus frequency.

### 2.3. Statistics

All statistical analyses were carried out using the statistical software R [32], version 4.0.5. We investigated the relationships of the variables HT, TL, SL, and TQ by simple correlation analyses. For this part, we relied on Spearman’s rank correlation coefficient, Rho (ρ).

The possible impact of the blood markers was assessed in two steps. First, we carried out simple correlation analyses for each marker with HT, TL, SL, and TQ, respectively. When the normality assumptions were violated (assessed via the Shapiro–Wilk test), we relied on Spearman’s rank correlation. For the Gaussian samples, we calculated the common correlation coefficient of Pearson. In this step, a correction of the resulting *p*-values for multiple comparisons was carried out by the method of Benjamini and Hochberg [33] to control the false discovery rate.

Second, we assessed the potential impact of each marker on the variables HT, TL, SL, and TQ, together with age and sex as covariates. To determine the best model for each of the response variables, we included age and sex as covariates in our linear models when a significant effect was detected. To find the best model, we followed a bottom-up approach. That is, we successively included the strongest predictors and their interaction in terms of *R*^2^. To decide which model is preferred, we mainly relied on the model selection criterion, BIC. When two models had an almost equal BIC, we considered the significance of the included terms as well as the *F*-tests for a model comparison. We checked the residual normality of the finally selected model by means of the Shapiro–Wilk test. When non-normality was suggested by the test, we repeated the analysis with a robust MM-type regression approach. For this regression type, we relied on the *lmrob* function from the *robustbase* package [34] with the estimation setting *KS2014,* as suggested by Koller and Stahel [35]. In this step, we also applied log transformations of the response variable and/or predictors when such a procedure was beneficial for the residual normality. We estimated the confidence intervals for the *R*^2^ statistic with the *boot* package [36,37] through a bias-corrected accelerated bootstrap with 2000 replications.

On a minor note, the variable HT has been recorded twice for each patient (left/right). This leads to a statistical setting with repeated measures. To ensure comparability of the results obtained for the different response variables (in particular, the *R*^2^), the results presented here rely on simple linear modeling, where the within-subject observations are considered independent. To ensure that this simplification was justified, we also considered the measuring side as the covariate. However, this covariate never showed a significant effect and therefore did not occur in the remainder of this paper. In addition, we verified our findings by repeating the analyses via linear mixed-effects models. All results obtained were identical; therefore, we refrain from presenting them here for the sake of readability.

We established the general significance level set at *p* < 0.05. The imputation of missing values was not carried out. Consequently, only the complete cases entered each respective analysis.

### 2.4. Ethical Approval

Ethical approval was obtained from Charité Universitätsmedizin Berlin (No: EA1/115/15).

## 3. Results

**Figure 3:**
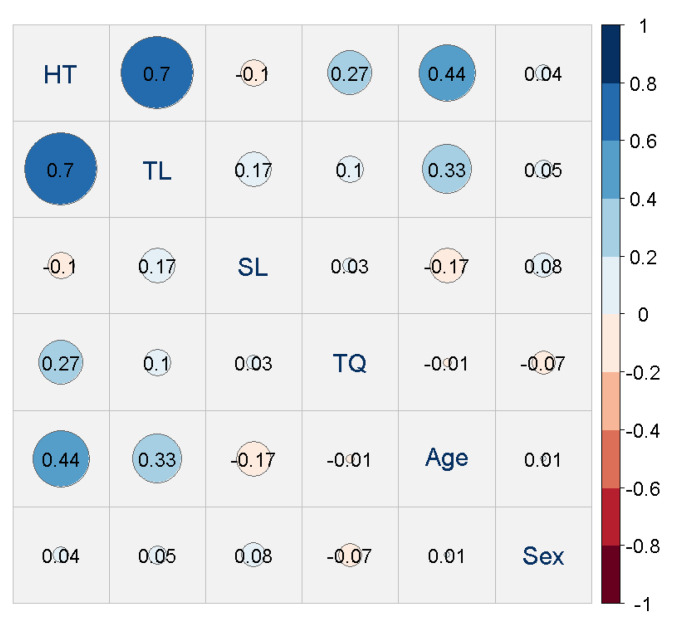
Correlation plot of audiological variables HT, TL, SL, the questionnaire score TQ, and the covariates age and sex (main diagonal of the plot). The off-diagonal elements of the plot visualize the correlation between these variables. For robustness and comparability, we entirely relied on Spearman’s rank correlation coefficient here and averaged the HT/TL values obtained from the left and right ears.

For a first understanding of our response variables HT, TL, SL, and TQ, and the covariates of sex and age, we illustrated the simple associations seen in Figure 3. This figure suggests that the pair HT/TL exhibits the highest association, followed by some margin by the pairs HT/age, TL/age, and HT/TQ. Furthermore, a sex effect seems absent.

The first step was to investigate potential links between HT, TL, SL, and TQ, respectively, and our recorded blood parameters were to be carried out by means of simple association measures as well. However, such an approach may lead to erroneous conclusions because the effects substantially change when taking the covariates of age and sex into account. Nevertheless, we present these results in Supplementary Information A (Supplementary Table S1) for the sake of completeness. In order to obtain our core results, we investigated the predictive power of the most promising blood parameters in a (robust) linear modeling framework, taking the covariate effects into account when present. The results are shown in the following.

### 3.1. Link of Hearing Threshold (HT) and Blood Markers

For the log-transformed HT, the best model incorporates sex, age, and vitamin D3, whereby the latter two also interact, resulting in an *R*^2^ of 26.9% (see Table 4 below). The strongest effect is age (*R*^2^ = 19.4), followed by vitamin D3 (additive increase to *R*^2^= 21.8) and sex (additive increase to *R*^2^ = 20.5). The interaction of age and vitamin D3 suggests that the coefficient of vitamin D3 is positive at younger ages, i.e., higher vitamin D3 levels are linked to a higher hearing threshold. This effect inverses approximately between the ages of 60 and 61. From this age on, the coefficient possesses a negative sign; thus, higher vitamin D3 levels are linked to lower hearing thresholds.

Recall from above that the TL is a strong predictor for HT. To ensure the stability of our estimation results, we also built a model similar to the one presented above but with TL as an additional predictor. This leads to a model for HT with TL, age, and vitamin D3 as the predictors, where the sex effect disappears. Nevertheless, the model interpretation remains comparable to the one with age as a predictor (see Supplementary Information B: Supplementary Table S2). Moreover, we verified that the interaction effect of age and vitamin D3 persists when more complex non-linear patterns are permitted. To achieve this, we followed a generalized additive modeling approach, which led to a similar interpretation (see also Supplementary Information B: Supplementary Table S3 and Supplementary Figure S1).

### 3.2. Link of Tinnitus Loudness (TL) and Blood Markers

The results for TL are different from those obtained previously for HT. More precisely, the strongest predictor for TL is age, and the corresponding univariate model achieves an *R*^2^ of 10.9%. Adding vitamin D3 to this model raises the *R*^2^ to 16.6%, indicating a contribution of vitamin D3 of approximately 5.7%. The estimated coefficients (Table 5 below) show an increase in TL with increasing age and with increasing vitamin D3 levels. Neither an interaction effect of the two variables nor a sex effect could be detected. Note that age and vitamin D3 are positively weakly but not significantly correlated (ρ = 0.148, *p* = 0.084).

Similar to the previous section (but with inversed roles), we also investigated the model’s stability by including HT as a predictor for TL. The corresponding results are very similar. In short, HT replaces age as the predictor, and the impact of vitamin D3 remains (see Supplementary Information B: Supplementary Table S4).

### 3.3. Link of Sensation Level (SL) and Blood Markers

The best model for the variable SL includes sex and uric acid, resulting in an *R*^2^ of 5.44% (see Table 6 below). In principle, only uric acid contributes to the *R*^2^ because a univariate model exclusively containing the sex variable attains less than 1% *R*^2^. This variable should, however, not be excluded because of the sex-specific differences in uric acid levels, which are not in a similar pattern for SL.

### 3.4. Link of Tinnitus Distress (TQ) and Blood Markers

In a regression model with covariates, none of the blood markers possesses clinically relevant predictive power for tinnitus distress (TQ). The strongest link was detected for erythrocytes, where an *R*^2^ of just 5% was reached when including sex as a covariate. Determining the exact contribution of the erythrocytes to the *R*^2^ is difficult because both sex and the blood marker are non-significant in the univariate models. Nevertheless, the estimated coefficients indicate that erythrocytes constitute the dominating variable (see Table 7 below).

**Figure 4:**
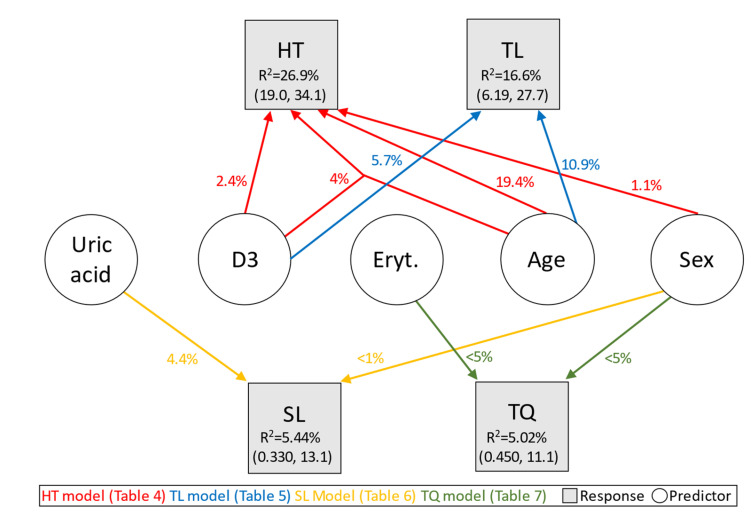
Contribution in terms of *R*^2^ (in %, with 95% confidence intervals for the selected models in parentheses) of significant blood markers and covariates for predicting HT (hearing threshold), TL (tinnitus loudness), SL (sensation level), and TQ (tinnitus distress). Simple arrows represent the contribution of a direct, additive effect in the best model. Joined arrows represent an interaction effect in the same model. D3—vitamin D3; Eryt.—erythrocytes.

Figure 4 below visualizes the results obtained so far from the models presented in this section. More precisely, it shows the contribution of the blood parameters erythrocytes and vitamin D3 as well as the covariates of age and sex to the *R*^2^ when predicting TQ, PSQ, TL, and HT, respectively.

## 4. Discussion

It is known that a self-assessment of tinnitus distress via psychological questionnaires involves other aspects of the tinnitus phenomenon than tinnitus loudness or sensation level. Even currently, there are no valid standards on how to measure and classify tinnitus or impairment due to tinnitus. Hearing status plays a critical role in experiencing tinnitus loudness (though not necessarily distress), even in individuals with mild to moderate hearing impairment [38].

Our simple correlation analyses of HT, SL, TL, TQ, age, and sex suggest an age dependency of both hearing ability and tinnitus loudness. Moreover, a positive correlation is present for both HT and TL, as well as HT and TQ. Sex does not seem to play a role in the correlation calculations. Tinnitus occurrence and hearing loss are closely linked [30,39]. Study results show that wearing hearing aids has a positive effect on the psyche, including tinnitus and the mental performance of those affected [31,40]. Especially with age, the hearing ability of many people also decreases. Hair cells and neuronal structures in the inner ear can degenerate, and in some people, the central processing of information in the brain becomes slower, leading to cognitive losses [41]. Moreover, the ability to discriminate speech also decreases with age, as higher frequencies and voices are no longer recognized or differentiated so well [42].

The absence of a correlation between tinnitus loudness and tinnitus distress is known from the literature [43]. In the assessment of tinnitus for clinical and research purposes, audiological parameters, such as hearing ability, tinnitus loudness, and sensation level, also play an indispensable role. This is especially true for the planning and evaluation of interventions.

When considering simple correlations, there seems to be a link between hearing ability and those blood parameters that indicate oxidative stress (IL6, Ferritin, SOD1, Q10, Creatinine, vitamin D3; see Supplementary Information A: Supplementary Table S1 for further details). Such stress possibly leads to an increased inflammation tendency, resulting in reduced hearing ability. There are many causes of hearing loss, and one of them could be oxidative stress because such stress and resulting inflammation may damage the inner ear [44]. This can contribute significantly to age- and noise-related hearing loss. Nevertheless, the detected correlations are small, and statistically, they are rather insignificant, particularly when considering the number of testing procedures.

In the regression models, age emerges as the most important predictor of hearing ability. Of the blood parameters, only vitamin D3 remains statistically relevant for HT prediction. A relation between hearing ability and vitamin D3 is described, e.g., in therapeutic approaches [45]. Furthermore, the effect is discussed in the context of otosclerosis [46] and the calcium transport system in the cochlea [47]. In this context, attempts to influence tinnitus via vitamin D3 administration have also been described [48]. Vitamin D deficiency in the ossicles leads to osteopenia, which can result in reduced hearing and, in some cases, even deafness. Vitamin D is therefore discussed as a very important vital substance for hearing. Higher vitamin D intake was also associated with reduced hearing difficulties [49]. In addition, several studies found that the incidence of hearing loss was increased with the lack of single micro-nutrients, such as vitamins A, B, C, D, and E, zinc, magnesium, selenium, iron, and iodine [50].

However, the studies are inconsistent, and, to our knowledge, D3 supplementation is not recommended in any national or international tinnitus guideline. This may well be related to the low explanatory power: in our model, vitamin D3 possesses only less than three percent explanatory power in terms of the *R*^2^. Furthermore, the relationship is not a simple linear trend but changes with age. More precisely, higher vitamin D3 concentrations were linked to higher hearing thresholds (worse hearing) in younger participants, whereas for older participants (approx. greater than 60 years old), the opposite effect was true (the higher the D3 level, the lower the hearing threshold, i.e., the better the hearing). It is noteworthy, however, that the vitamin D3 levels do not exceed the reference range for our sample. Consequently, we can assume that excessive supplementation of vitamin D3 can lead to vitamin D toxicity (VDT), which is linked to hypercalciuria, hypercalcemia, elevated 25(OH)D > 150 ng/mL (>375 nmol/L), and may result, among others, in hearing loss [51], which does not occur for our patients. Another study [52] confirmed the potential effect of vitamin D3 supplementation on the THI scores in vitamin D3-deficient tinnitus patients. Again, this and our study cannot be directly compared since our participants were not vitamin D3 deficient. This is, however, possibly the effect of supplementation, which we did not control for.

In conclusion, a hearing-protective additional intake of D3 may be considered individually for older people. In any case, the expected therapeutic effect is marginal.

Exposures to ototoxic and cytotoxic drugs are recognized as factors that increase the risk of developing tinnitus. These factors are known to cause the death of cochlear hair cells by apoptosis rather than necrosis [53]. Research is therefore indicated to discover the agents that can block apoptosis. The action of antioxidants is attracting the most attention, with vitamins or magnesium showing initial promise [54]. Further, it has been suggested that a vitamin D (25(OH)D) deficiency may be correlated with tinnitus annoyance and with tinnitus loudness [55]. In their study, the authors observed that the 25(OH)D level was lowered for tinnitus patients compared to healthy controls. Moreover, within the tinnitus group, higher scores on the VAS loudness scale, as well as higher annoyance, which was measured by the THI questionnaire, were associated with lower levels of this indicator. It should be noted that, in our study, the vitamin D3 level was within the reference range, and tinnitus loudness was determined by the tinnitus matching procedure, suggesting that these two studies cannot be directly compared.

Through our modeling approach, we observed that both age and the vitamin D3 level are positively related to tinnitus loudness, not tinnitus annoyance per se (see Section 3.4, where no link between the TQ values and vitamin D3 was found). The limitations described above apply here as well. In particular, we were unable to control for supplementation.

Research suggests that inflammation and increased oxidative stress, produced by hyperuricemia (in gout), can impact the auditory system [56]. Otoacoustic emission studies suggest that subclinical changes in cochlear function are associated with hyperuricemia. It was postulated that hyperuricemia is associated with increased stiffness and/or impaired blood supply to the outer hair cells, impairing their electromotor response [57]. The vascular impairment associated with increased uric acid levels may be a risk factor for age-related hearing loss [58]. In a case-control study of sudden sensorineural hearing loss, blood glucose, HbA1C, uric acid, factor VIII, and homocysteine were significantly associated with disease severity in SSHL patients [59], while Wen-yan et al. found contradicting results regarding the uric acid levels [60]. In a representative study of adults in the USA, elevated uric acid levels were found to be inversely associated with hearing thresholds in pure-tone audiometry [61]. Moreover, already in 1998, it was hypothesized that idiopathic tinnitus of intermittent intensity might be related to hyperuricemia [62].

Our results indicate a small influence of uric acid on the measured sensation level of tinnitus. This should, however, not be over-interpreted given the comparably low predictive power (less than 5% contribution to the overall *R*^2^) on the one hand. On the other hand, the sign of our effect points towards a decrease in SL with increasing uric acid levels, which should not play a role in dietary advice.

Although the biomarkers associated with stress have been extensively described [9,30,63,64], no established biomarkers of tinnitus distress have been identified thus far. Two of the forty-nine studies included in the meta-analysis from 2021 [11] on biomarkers of tinnitus comprised measurements of the erythrocyte levels. Resuli [65] demonstrated that the erythrocyte and serum ALP levels were significantly associated with tinnitus in children exposed to cigarette smoke. The association of red blood cell counts (and alkaline phosphatase levels/urine cotinine) in smokers with tinnitus was observed by Lee and Kim [66]. Other studies on red blood cell counts, which were included in another recent review [25], did not show significant results.

For the prediction of TQ, we observed that neither sex nor any of the blood markers possessed a predictive power in a univariate setting. However, when including both sex and erythrocytes as predictors in a model, a small (joint *R*^2^ < 5%) but significant effect was observed. In this context, note that the erythrocyte levels differed significantly between men and women (1.52 pL in women vs. 1.63 pL in men, *p* < 0.0001). Hence, the effect of the sex variable may most likely result from the sex-specific difference in the erythrocytes. In addition, we could not detect a significant age effect on tinnitus distress, as it has been observed in previous studies (see, e.g., [67]). This may, however, very well result from the limited power due to our much lower sample size (200 vs. 1180). The same argument could very well also explain why our results show a very limited influence of sex on the TQ scores. This is not in line with previous research [68,69], which described the presence of general sex-driven differences in the experience of stress.

We also investigated whether the link between erythrocytes and tinnitus distress remains when including perceived stress as an additional covariate to specify the statements on the connection between the blood parameters and this variable in tinnitus patients (see [9]). In short, in such a model, the PSQ is by far the strongest predictor for the TQ, with an *R*^2^ of almost 37.8%, highlighting the importance of considering construct overlap. Whilst erythrocytes, age, and sex also enter the model, they possess only little predictive power (all <2% *R^2^*) (see Supplementary Information C: Supplementary Table S5 and Supplementary Figure S2 for details).

In addition to the statistical analyses presented in this paper, we also conducted a multivariate regression approach for each of the variables, HT, TL, SL, and TQ. More specifically, we relied on an elastic net regression with all the blood markers and the covariates of age and sex as predictors to investigate the possibility that multiple markers jointly possess predictive power. However, compared to the analyses presented in this paper, the results of the elastic net approach (not shown) did not reveal any supplementary information. In particular, no additional clinically relevant impact of the blood markers was discovered.

Another limitation also results from the fact that we investigated the effects of erythrocytes, uric acid, and vitamin D3 when considering the covariates, although, in principle, these effects vanish when correcting the resulting *p*-values for multiple comparisons. Together with the low contribution to the *R*^2^, this underlines that these blood marker effects should be considered with caution. In order to avoid such difficulties and to better determine effect sizes, further investigations via a controlled experiment would be needed. In addition, we considered only age and sex as covariates and could have neglected other important variables (e.g., education or social support).

In any case, given the low predictive power of even our selected blood parameters, one may consider drastically increasing sample sizes in future studies (as illustrated by the large confidence intervals of the estimated *R*^2^ values in Figure 4).

## 5. Conclusions

Our paper confirms the necessity of measuring tinnitus via audiological parameters (e.g., sensation level, tinnitus loudness, hearing thresholds) and distress-related questionnaires, depending on the research question investigated. With the multi-dimensionality of the tinnitus construct, the main concern of this work was to determine possible biomarkers for diagnostics and therapy of tinnitus via the blood parameters. However, in our sample, the predictive power of the blood parameters on tinnitus-related impairments is, in principle, of limited clinical relevance. For tinnitus distress, the erythrocytes seem to be an important factor as a hematological inflammation marker. A different study design could help quantify a potential relationship between tinnitus distress and erythrocytes.

For hearing as well as the closely related tinnitus loudness, vitamin D3 appears to be a factor of interest in our data in combination with age. The tinnitus sensation level could vary in intensity due to increased uric acid levels. For clinical practice, it can be recommended, especially in older patients, a controlled supplementation of vitamin D3 could be considered a hearing-protective procedure.

## Figures and Tables

**Table 1:** Summary of studies on tinnitus and blood markers.

I. Immune activity	
Cellular immune response	Increase ↑: MPV (related to HFHL) [12], PDW, PC, 11-dTxB2 [13]
Inflammation direct	Increase ↑: IL-1α (related to tonal tinnitus), IL-1, IL-2, TNF-α [14,15] Decrease ↓: IL-10, HSP-70, CD16NK, CD19 (related to ACTH) [16]
Inflammation indirect	Increase ↑: NLR (related to HFHL-TN), PLR, CRP, ESR [17,18]
II. Oxidative parameters	
	Increase ↑: GST, GSH, MDA [19], TAS, TOS OSI [20,21]
III. Metabolism	
Lipid metabolism	Increase ↑: TC, TG, or TRG, LDL [22] Decrease ↓: HDL [23]

The term “Increase ↑” stands for an increase in, e.g., tinnitus distress (or the number of tinnitus patients, depending on the study) with increasing levels of the corresponding biomarker. “Decrease ↓” is interpreted analogously.

**Table 2:** Measured blood markers with units and basic descriptive statistics.

Marker (Unit)	Female (*N* = 102) Mean (± St. Dev.)	Male (*N* = 98) Mean (± St. Dev.)	Total (*N* = 200) Mean (± St. Dev.)
Basophils (absolute/nL)	0.0443 (±0.0204)	0.0523 (±0.0242)	0.0482 (±0.0226)
Eosinophils (absolute/nL)	0.125 (±0.0875)	0.158 (±0.111)	0.141 (±0.101)
Immat. gran. (absolute/nL)	0.0220 (±0.0134)	0.0284 (±0.0196)	0.0251 (±0.0170)
Leukocytes (absolute/nL)	6.48 (±1.51)	6.82 (±1.88)	6.65 (±1.71)
Lymphocytes (absolute/nL)	2.00 (±0.647)	1.97 (±0.561)	1.98 (±0.606)
Monocytes (absolute/nL)	0.481 (±0.148)	0.568 (±0.170)	0.523 (±0.164)
Neutrophils (absolute/nL)	3.83 (±1.22)	4.02 (±1.47)	3.93 (±1.34)
NLR (ratio)	2.12 (±0.984)	2.17 (±0.909)	2.14 (±0.946)
IL6 (ng/L)	2.88 (±11.8)	2.12 (±1.69)	2.51 (±8.53)
TNF-α (pg/mL)	0.511 (±1.95)	0.348 (±0.329)	0.431 (±1.41)
CRP (mg/L)	1.88 (±2.29)	1.79 (±2.40)	1.83 (±2.34)
Ferritin (μg/L)	88.5 (±57.4)	177 (±114)	132 (±99.9)
Fibrinogen (g/L)	2.72 (±0.581)	2.72 (±0.568)	2.72 (±0.573)
MPV (fl)	10.6 (±0.899)	10.7 (±1.09)	10.7 (±0.998)
Thrombocytes (nL)	256 (±58.9)	232 (±46.5)	244 (±54.3)
Erythrocytes (pL)	4.56 (±0.331)	5.10 (±0.361)	4.83 (±0.439)
Haematocrit (L/L)	0.404 (±0.0258)	0.449 (±0.0279)	0.426 (±0.0351)
Hemoglobin (g/dL)	13.6 (±0.771)	15.3 (±0.994)	14.4 (±1.24)
MCH (pg)	29.8 (±1.31)	30.0 (±1.35)	29.9 (±1.33)
MCHC (g/dL)	33.6 (±0.930)	34.0 (±0.915)	33.8 (±0.944)
MCV (fL)	88.6 (±3.49)	88.1 (±3.70)	88.3 (±3.60)
RDW-CV (%)	12.9 (±0.904)	12.8 (±0.590)	12.8 (±0.768)
Lipid Peroxidase (μmol/L)	110 (±158)	40.8 (±59.2)	75.8 (±125)
SOD1 (ng/mL)	63.3 (±2.25)	68.0 (±19.3)	65.6 (±13.8)
SOD2 (ng/mL)	52.7 (±13.6)	65.4 (±14.8)	58.9 (±15.5)
Q10 (μmol/mmol)	0.220 (±0.0617)	0.250 (±0.0793)	0.235 (±0.0722)
Cholesterol (mg/dL)	219 (±40.7)	207 (±39.3)	213 (±40.4)
HDL (mg/dL)	73.2 (±18.0)	52.8 (±11.8)	63.2 (±18.3)
LDL (mg/dL)	138 (±37.8)	138 (±34.6)	138 (±36.2)
Lipoprotein (a) (nmol/L)	59.6 (±80.5)	38.0 (±55.7)	49.0 (±70.1)
Non-HDL (mg/dL)	146 (±42.0)	154 (±39.3)	150 (±40.8)
Triglyceride (mg/dL)	109 (±53.3)	142 (±71.8)	126 (±65.0)
Albumin (g/L)	46.3 (±2.38)	46.4 (±2.63)	46.4 (±2.50)
Gamma-GT (U/L)	19.8 (±11.2)	32.0 (±18.4)	25.8 (±16.3)
GOT (U/L)	22.8 (±5.23)	28.3 (±10.7)	25.5 (±8.78)
GPT (U/L)	22.8 (±10.1)	35.4 (±21.0)	29.0 (±17.6)
Creatinine (mg/dL)	0.755 (±0.0967)	0.954 (±0.136)	0.852 (±0.154)
GFR (ml/min)	84.9 (±7.94)	83.9 (±8.99)	84.4 (±8.46)
Uric acid (mg/dL)	4.21 (±0.923)	6.13 (±3.14)	5.15 (±2.49)
Calcium (mmol/L)	2.34 (±0.0985)	2.34 (±0.0805)	2.34 (±0.0899)
Magnesium (mmol/L)	0.842 (±0.0543)	0.849 (±0.0541)	0.845 (±0.0542)
Selenium (μmol/L)	1.02 (±0.220)	1.01 (±0.239)	1.01 (±0.229)
Vitamin D3 (nmol/L)	67.6 (±25.8)	66.9 (±24.0)	67.2 (±24.9)
Zinc (μmol/L)	12.4 (±1.67)	13.0 (±2.03)	12.7 (±1.88)
D-dimer (mg/L)	0.386 (±0.502)	0.435 (±1.31)	0.410 (±0.985)
Telomere length (ratio)	1.15 (±0.248)	1.12 (±0.267)	1.14 (±0.257)

CRP = C-reactive protein; dL = deciliter; fL = femtolitre; g= gram; GFR = glomerular filtration rate; GOT = glutamat-oxalacetat-transaminase; GPT = glutamat-pyruvat-transaminase; IL6 = interleukin-6; L = liter; MCH = mean corpuscular/cellular hemoglobin; MCHC = mean corpuscular hemoglobin concentration; MCV = mean corpuscular/cell volume; mg = milligram; mmol = millimole; MPV = mean platelet volume; ng = nanogram; nL = nanoliter; nmol = nanomole; NLR = neutrophil-lymphocyte-ratio; pg = picogram; pL = picolitre; RDW_CV = red blood cell distribution width; TNF-α = tumor necrosis factor-alpha; U = unified atomic mass unit; μg = microgram; μmol = micromole.

**Table 3:** Sample characteristics and questionnaires (*N = 200*).

Variable	Total (*N* = 200) Mean (± St. Dev.)/Freq. (Prop.)
HT (right)	26.3 (±13.6) dB
HT (left)	27.6 (±13.0) dB
TF (right) Not measured TF (left) Not measured TL	6040 (±2260) Hz 99 (49.5%) 5760 (±2550) Hz 94 (47.0%) 47.3 (±20.4) dB
Not measured	62 (31.0%)
SL	6.59 (±5.71) dB
Not measured	62 (31.0%)
TQ	43.8 (±19.0)
Age	54.7 (±8.44) years
Sex	
Female	102 (51%)
Male	98 (49%)

Empirical mean (standard deviation) and absolute frequencies (proportions) for numerical and categorical variables, respectively.

**Table 4:** Estimation results for the best model for predicting HT with blood markers.

Term	Estimate	Std. Error	*t*-Statistic	*p*-Value
(Intercept)	−7.11	1.73	−4.10	<0.001
Age	0.17	0.03	5.38	<0.001
Log (Vitamin D3)	2.14	0.42	5.06	<0.001
Sex	0.13	0.05	2.86	0.005
Age:log (Vitamin D3)	−0.04	0.01	−4.57	<0.001

**Table 5:** Estimation results for the best model for predicting TL with blood markers.

Term	Estimate	Std. Error	*t*-Statistic	*p*-Value
(Intercept)	−48.44	20.15	−2.40	0.018
Age	0.74	0.20	3.79	<0.001
Log (Vitamin D3)	13.52	4.47	3.02	0.003

**Table 6:** Estimation results for the best model for predicting SL with blood markers.

Term	Estimate	Std. Error	*t*-Statistic	*p*-Value
(Intercept)	11.95	2.67	4.47	<0.001
Sex	2.65	1.14	2.32	0.022
Log (Uric acid)	−4.46	1.80	−2.48	0.014

**Table 7:** Estimation results for the best model for predicting TQ with blood markers.

Term	Estimate	Std. Error	*t*-Statistic	*p*-Value
(Intercept)	−43.15	30.70	−1.41	0.161
Erythrocytes	58.60	20.21	2.90	0.004
Sex	−9.76	3.65	−2.67	0.008

## Data Availability

The data presented in this study are available on request from the corresponding author. The data are not publicly available due to institutional rules.

## Supplementary Materials

The following supporting information can be downloaded at: https://www.mdpi.com/article/10.3390/jcm12113814/s1, Table S1: Correlation of blood parameters with HT, TL, SL, and TQ. From left to right, the columns show the name of the correlated variable, the correlated blood parameter, the correlation test carried out (P: Pearson, S: Spearman), the original and adjusted *p*-value, and the (rank-)correlation coefficient as effect size. Table S2: Estimation results for the best model for predicting HT with TL and blood markers. Table S3: Estimation results for the GAM predicting HT with blood markers. Part (a.) of the table shows the estimation results for the parametric model component, part (b.) the results corresponding to the non-parametric model component. Table S4: Estimation results for the best model for predicting TL with HT and blood markers. Table S5: Estimation results for the best model for predicting TL with PSQ and biomarkers. Figure S1: Visualization of the estimated effects in the GAM. The two top and bottom right panels show the single-variable-effects, the bottom left panel visualizes the interaction effect. *D3*—vitamin D3; *s()*, *ti()*, *f()*—specification of the smooth terms. Figure S2: Contribution in terms of *R^2^* (in %) of significant blood markers and covariates (including PSQ) for predicting TQ. Refs. [70,71,72,73] are cited in the Supplementary Materials File.
