# Supplementary material for: Limited Link of Common Blood Parameters with Tinnitus
Source: J Clin Med. 2023 Jun 1;12(11):3814. doi: 10.3390/jcm12113814 (PMC10253676; doi:10.3390/jcm12113814)
Supplement: Supplementary file 1 [file jcm-12-03814-s001.zip › jcm-2377716-supplementary.pdf]

Journal of
Clinical Medicine

MDPI

Supplementary Information - Limited link of common blood parameters with tinnitus

Supplementary Information A – Simple correlations of blood markers with HT, TL, SL, and TQ

Supplementary Table S1 shows all those blood markers subject to significant correlation with at least one of the correlated variables (column *p*-value). However, only a few of the potentially significant associations remain significant after correcting for multiple comparisons (adj. *p*-value). Moreover, effect sizes are comparably small for all blood markers.

Supplementary Table S1. Correlation of blood parameters with HT, TL, SL, and TQ. From left to right, the columns show the name of the correlated variable, the correlated blood parameter, the correlation test carried out (P: Pearson, S: Spearman), the original and adjusted *p*-value, and the (rank-)correlation coefficient as effect size.

Variable	Biomarker	Test	*p*-value	Adj. *p*-value	Corr. coeff.
HT	IL6	S	0.029	0.213	0.154
HT	Ferritin	S	0.010	0.119	0.181
HT	SOD1	S	0.023	0.207	-0.161
HT	Q10	S	<0.001	0.019	0.235
HT	GFR	S	<0.001	0.017	-0.249
HT	Creatinine	S	0.037	0.213	0.148
HT	Vitamin D3	S	0.005	0.082	0.196
HT	Telomere length	S	0.034	0.213	-0.150
TL	IL6	S	0.045	0.513	0.171
TL	SOD2	P	0.021	0.450	-0.196
TL	Q10	P	0.029	0.450	0.186
TL	Vitamin D3	P	<0.001	0.046	0.277
SL	-	-	-	-	-
TQ	Immat. gran.	S	0.049	0.372	0.139
TQ	Leukocytes	S	0.018	0.318	0.168
TQ	Neutrophils	S	0.044	0.372	0.142
TQ	Thrombocytes	S	0.016	0.318	0.170
TQ	RDW-CV	S	0.021	0.318	0.163
TQ	SOD1	S	0.028	0.318	-0.156

Supplementary Information B – Stability of vitamin D3 effects

A strong association exists between HT and TL. Therefore, the two variables are a canonical choice for predicting each other. A natural question arising is whether the Vitamin D3 effects remain when TL is included as potential predictor of HT, and vice versa. This leads to the regression models presented in the following.

Prediction of HT: Inclusion of TL as covariate when predicting the log-transformed HT in a robust regression setting leads to the results shown in the table below. The strongest predictor is TL with an R^2 of 49.2%. Adding the other potential covariates leads to results comparable to those obtained in the main text, with the exception that sex does not play a role anymore. The final model (see Supplementary Table S2 below) also includes an interaction of age and Vitamin D3 and reaches an R^2 of 58.5%. The estimated coefficients suggest a reduction of HT with increasing vitamin D3 from approximately age 55 on.

Supplementary Table S2. Estimation results for the best model for predicting HT with TL and blood markers.

Term	Estimate	Std. Error	*t*-statistic	*p*-value
(Intercept)	-4.01	1.65	-2.43	0.016
TL	0.01	0.00	13.24	<0.001
Age	0.12	0.03	3.72	<0.001
log(Vitamin D3)	1.38	0.41	3.38	<0.001
Age:log(Vitamin D3)	-0.02	0.01	-3.21	0.001

Furthermore, we investigated the interaction effect of age and vitamin D3 in the model predicting HT in more detail, because it is possible that more complex patterns are present. Such patterns, e.g., non-linearities, could not be easily detected by a simple linear model. To take a rather general approach, we fitted a so-called generalized additive model (GAM) for predicting HT. We selected the strongest predictors from the comparable linear model as predictors as well. The following Supplementary Table S3 shows the estimation results of the resulting GAM.

Supplementary Table S3. Estimation results for the GAM predicting HT with blood markers. Part a.) of the table shows the estimation results for the parametric model component, part b.) the results corresponding to the non-parametric model component.

a.)

Term	Estimate	Std. Error	*t*-statistic	*p*-value
(Intercept)	3.11	0.03	99.18	<0.001
Sex	0.13	0.04	2.78	0.006

b.)

Term	Edf	Ref. df	*t*-statistic	*p*-value
s(Age)	2.84	9.00	10.25	<0.001
s(Vitamin D3)	0.90	9.00	0.76	0.004
ti(Age,Vitamin D3)	6.12	16.00	2.93	<0.001

From these results it is visible that all variables remain significant as well when allowing for non-linear dependence patterns. Moreover, as shown in Supplementary Figure S1 below (bottom left panel), low HT values are observed for low vitamin D3 levels at young age, and high vitamin D3 levels at high age. This confirms the results of the simple linear model.

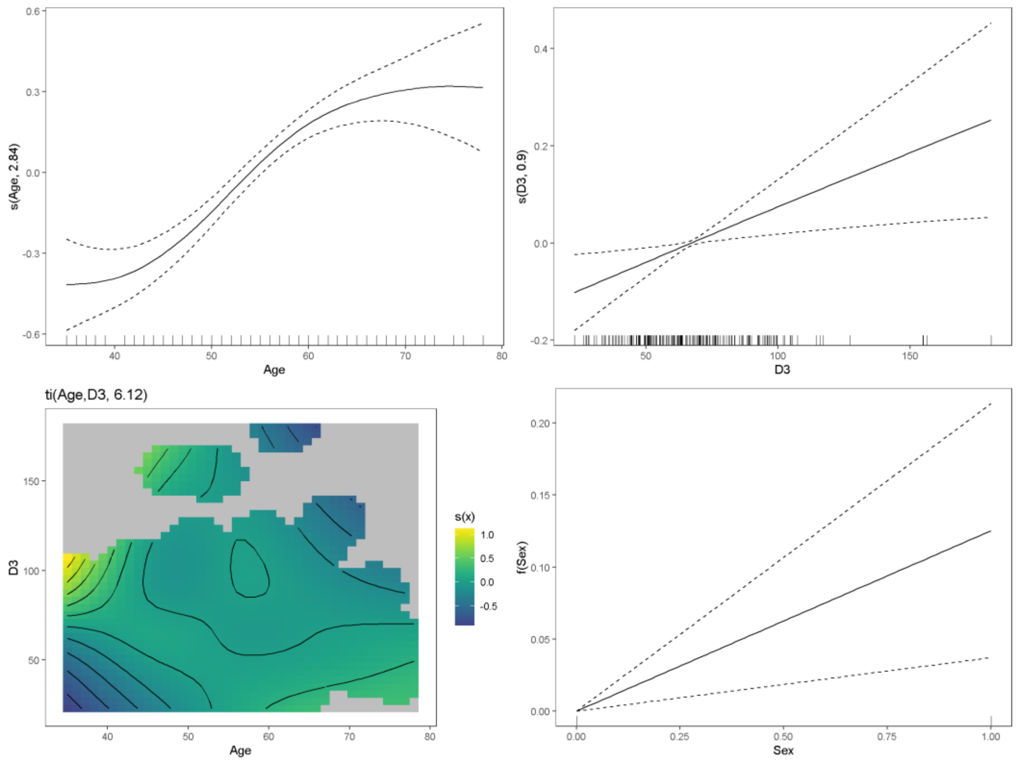

Supplementary Figure S1. Visualization of the estimated effects in the GAM. The two top and bottom right panels show the single-variable-effects, the bottom left panel visualizes the interaction effect. *D3* – vitamin D3; *s()*, *ti()*, *f()* – specification of the smooth terms.

Prediction of TL: When including HT as predictor instead of age a robust regression leads to the results in the Supplementary Table S4 below. The impact of vitamin D3 is again comparably small: a model with only HT as predictor reaches an R^2 of 45.4%, adding vitamin D3 increases the R^2 to 50.0%.

Supplementary Table S4. Estimation results for the best model for predicting TL with HT and blood markers.

Term	Estimate	Std. Error	*t*-statistic	*p*-value
(Intercept)	-59.80	10.57	-5.66	<0.001
log(HT)	27.25	1.82	14.96	<0.001
log(Vitamin D3)	5.05	2.54	1.99	0.048

Supplementary Information C – Stability of erythrocytes effect

A potential strong driver for TQ is general stress (PSQ) and other psychological variables like depression, anxiety, personality traits or pain [1–4]. Moreover, our results show that erythrocytes possess a limited, but significant predictive power on TQ. To check whether a similar pattern is present for only tinnitus-related distress, we investigated a couple of robust linear models where PSQ was introduced as potential covariate in addition to age and sex. Supplementary Table S5 shows the results of the preferred model.

Supplementary Table S5. Estimation results for the best model for predicting TL with PSQ and biomarkers.

Term	Estimate	Std. Error	t-statistic	p-value
(Intercept)	-92.37	24.74	-3.73	<0.001
PSQ	0.93	0.08	11.11	<0.001
Sex	-6.21	2.76	-2.25	0.026
Erythrocytes	47.50	15.21	3.12	0.002

Age	0.33	0.13	2.54	0.012

In terms of R^2, PSQ is the strongest predictor achieving 37.8% alone. The second and third strongest predictors are age (raise to R^2=40.2%) and erythrocytes (further raise to R^2=41.6%), respectively. The final model including sex as covariate as well reaches an R^2 of 43.0%, the inclusion of further covariates is not supported (see Supplementary Figure S2 below). Hence, erythrocytes possess a small but significant predictive power for purely tinnitus-related distress as well.

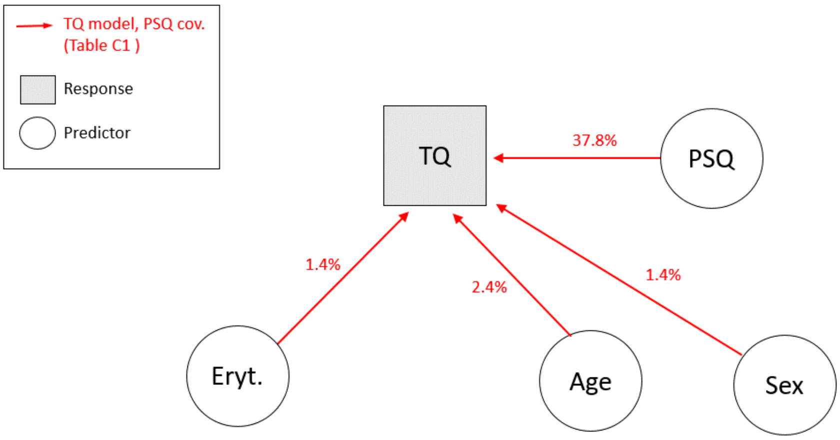

Supplementary Figure S2. Contribution in terms of R^2 (in %) of significant blood markers and covariates (including PSQ) for predicting TQ.

References

1. Basso, L.; Boecking, B.; Brueggemann, P.; Pedersen, N.L.; Canlon, B.; Cederroth, C.R.; Mazurek, B. Chapter 3—Subjective hearing ability, physical and mental comorbidities in individuals with bothersome tinnitus in a Swedish population sample. In *Progress in Brain Research*; Schlee, W., Langguth, B., Kleinjung, T., Vanneste, S., De Ridder, D., Eds.; Elsevier: Amsterdam, The Netherlands, 2021; Volume 260, pp. 51–78.
2. Boecking, B.; Rose, M.; Brueggemann, P.; Mazurek, B. Two birds with one stone.–Addressing depressive symptoms, emotional tension and worry improves tinnitus-related distress and affective pain perceptions in patients with chronic tinnitus. *PLoS ONE* **2021**, *16*, e0246747, https://doi.org/10.1371/journal.pone.0246747.
3. Brueggemann, P.; Mebus, W.; Boecking, B.; Amarjargal, N.; Niemann, U.; Spiliopoulou, M.; Dobel, C.; Rose, M.; Mazurek, B. Dimensions of Tinnitus-Related Distress. *Brain Sci.* **2022**, *12*, 275, https://doi.org/10.3390/brainsci12020275.
4. Niemann, U.; Brueggemann, P.; Boecking, B.; Mebus, W.; Rose, M.; Spiliopoulou, M.; Mazurek, B. Phenotyping chronic tinnitus patients using self-report questionnaire data: cluster analysis and visual comparison. *Sci. Rep.* **2020**, *10*, 1–10, https://doi.org/10.1038/s41598-020-73402-8.
